# Identification and Characterization of New *RNASEH1* Mutations Associated With PEO Syndrome and Multiple Mitochondrial DNA Deletions

**DOI:** 10.3389/fgene.2019.00576

**Published:** 2019-06-14

**Authors:** Lidia Carreño-Gago, Cora Blázquez-Bermejo, Jordi Díaz-Manera, Yolanda Cámara, Eduard Gallardo, Ramon Martí, Javier Torres-Torronteras, Elena García-Arumí

**Affiliations:** ^1^Departament de Patologia Mitocondrial i Neuromuscular, Hospital Universitari Vall d’Hebron Institut de Recerca (VHIR), Universitat Autònoma de Barcelona, Barcelona, Spain; ^2^Centro de Investigación Biomédica en Red de Enfermedades Raras (CIBERER), Instituto de Salud Carlos III, Barcelona, Spain; ^3^Servei de Neurologia, Malalties Neuromusculars, Hospital de la Santa Creu i Sant Pau i Institut de Recerca de HSCSP, Universitat Autònoma de Barcelona, Barcelona, Spain; ^4^Àrea de Genètica Clínica i Molecular, Hospital Universitari Vall d’Hebron, Barcelona, Spain

**Keywords:** mtDNA, mitochondrial disease, PEO, multiple mtDNA deletions, *RNASEH1*

## Abstract

Mitochondrial DNA (mtDNA) depletion and deletion syndrome encompasses a group of disorders caused by mutations in genes involved in mtDNA replication and maintenance. The clinical phenotype ranges from fatal infantile hepatocerebral forms to mild adult onset progressive external ophthalmoplegia (PEO). We report the case of a patient with PEO and multiple mtDNA deletions, with two new homozygous mutations in *RNASEH1*. The first mutation (c.487T>C) is located in the same catalytic domain as the four previously reported mutations, and the second (c.258_260del) is located in the connection domain, where no mutations have been reported. *In silico* study of the mutations predicted only the first mutation as pathogenic, but functional studies showed that both mutations cause loss of ribonuclease H1 activity. mtDNA replication dysfunction was demonstrated in patient fibroblasts, which were unable to recover normal mtDNA copy number after ethidium bromide-induced mtDNA depletion. Our results demonstrate the pathogenicity of two new *RNASEH1* variants found in a patient with PEO syndrome, multiple deletions, and mild mitochondrial myopathy.

## Introduction

The mitochondrion is a cellular organelle crucial for cell metabolism that integrates various metabolic pathways, including oxidative phosphorylation (OXPHOS), fatty acid oxidation, Krebs cycle, urea cycle, gluconeogenesis, and ketogenesis (Gorman et al., 2016). Mitochondrial function is under the control of two genomes, nuclear DNA (nDNA) and mitochondrial DNA (mtDNA). mtDNA is a 16.6-kb circular and double-stranded DNA molecule that encodes 2 rRNAs, 22 tRNAs, and 13 peptides from OXPHOS complexes I, III, IV, and V. However, most mitochondrial proteins are encoded by nDNA genes (Anderson et al., 1981). Nuclear-encoded mitochondrial proteins are synthetized in the cytosol and transported into the mitochondria where they perform their functions (Herrmann et al., 2012). A large number of these mitochondrial proteins are OXPHOS complex subunits or complex assembly factors and therefore, they are directly involved in the electron transport chain and energy production in the form of ATP. Furthermore, a group of nuclear genes are involved in several mitochondrial processes, such as mtDNA maintenance, transcription and translation, and control of mitochondrial dynamics, which are also essential for proper OXPHOS function. When a malfunction occurs in one of these proteins, the mitochondrial OXPHOS system is unable to produce sufficient energy to maintain cell requirements, and this deficit leads to a number of mitochondrial diseases (Wallace, 1999). Therefore, mitochondrial diseases are caused by mutations in mtDNA genes and in a very long list of nuclear mitochondrial-related genes (Cerritelli et al., 2003; Ruhanen et al., 2011; Uhler and Falkenberg, 2015).

Multiple mtDNA genome copies are present in cells to provide the components for energy production. mtDNA replication is unsynchronized with the cell cycle as it occurs in dividing and postmitotic cells. Correct mtDNA maintenance requires adequate expression of a number of nuclear-encoded proteins involved in mtDNA replication, mitochondrial deoxynucleotide pool synthesis, mitochondrial dynamics, and other proteins with uncertain function. Changes in these genes are associated with secondary mtDNA changes, such as mtDNA deletions or depletion, which cause OXPHOS dysfunction and result in a group of disorders known as mitochondrial depletion and deletion syndromes (MDDSs). The clinical phenotype associated with MDDSs ranges from fatal infantile hepatocerebral forms to mild adult-onset syndromes, such as progressive external ophthalmoplegia (PEO) (Suomalainen and Kaukonen, 2001; El-Hattab et al., 2017).

The human *RNASEH1* gene encodes an endonuclease involved in DNA replication in both the nucleus and mitochondria. Ribonuclease H1 (RNase H1) is the only known ribonuclease of this type in the mitochondria (Al-Behadili et al., 2018; Rusecka et al., 2018) and, in particular, this enzyme is implicated in the RNA degradation of the double-stranded RNA-DNA hybrid created to prime DNA synthesis during mtDNA replication (Cerritelli et al., 2003; Ruhanen et al., 2011; Uhler and Falkenberg, 2015; Al-Behadili et al., 2018). *RNASEH1* mutations have been described in patients with clinical MDDS associated with adult-onset PEO and multiple mtDNA deletions (Reyes et al., 2015; Bugiardini et al., 2017; Sachdev et al., 2018). The predominant clinical traits of patients with *RNASEH1* mutations are PEO, ptosis, dysphagia, facial and/or proximal weakness, ataxia, and respiratory impairment.

To date, only four mutations in *RNASEH1*, all occurring in the catalytic RNase H1 domain, have been identified in a total of 14 patients with a mild clinical phenotype (Reyes et al., 2015; Bugiardini et al., 2017; Sachdev et al., 2018). Here, we report the case of a patient diagnosed with mild mitochondrial myopathy characterized by PEO and multiple mtDNA deletions caused by two previously unreported mutations in the *RNASEH1* gene, one located in the catalytic domain and the other in the RNase H1 connection domain.

## Materials and Methods

All the experimental protocols were performed with appropriate informed consent and approval of the Clinical Research Ethics Committee of the Hospital Universitari Vall d’Hebron (PR(IR)66/2016).

### Histopathological Studies

A skeletal muscle biopsy from the left biceps was performed. Five-micron sections of frozen muscle were double-stained with cytochrome c oxidase (COX) and succinate dehydrogenase (SDH) (Dubowitz and Sewry, 2007).

### Cell Culture Conditions

Patient fibroblasts and fibroblasts from healthy age-matched donors were obtained by skin biopsy and used in the study. Fibroblasts were grown in high glucose (4.5 g/L) DMEM (Gibco) supplemented with 10% FBS, 200 mM L-glutamine, 100 mM sodium pyruvate 100 U/mL penicillin and 0.1 mg/mL streptomycin. In order to force OXPHOS for ATP production, in some experiments cells were grown in DMEM without glucose (Gibco) supplemented with 10% FBS, 1 g/L galactose, 200 mM L-glutamine, 100 mM sodium pyruvate, 100 U/mL penicillin and 0.1 mg/mL streptomycin.

To induce mtDNA depletion in fibroblasts, the same number of cells was seeded and cultured to confluence (day 0). Fibroblasts were then treated with 15 ng/mL ethidium bromide (EtBr) for 4 days. After EtBr was withdrawn (day 4), cells were kept in culture media for 10 additional days (day 14). Cells were collected on days 0, 2, 4, 7, 9, and 14, and maintained frozen at -20°C until further DNA extraction.

### Genetic Studies

We sequenced the exonic and intron flanking regions of 17 nuclear genes (*DGUOK, DNA2, FBXL4, MGME1, MFN2, MPV17, OPA1, POLG, POLG2, RNASEH1, RRM2B, SLC25A4, SUCLA2, SUCLG1, SPG7, TK2*, and *TWNK*) involved in mtDNA replication and maintenance by next-generation sequencing, using a previously designed panel with GeneRead Custom Panel V.2 (Qiagen) technology. Libraries were prepared with the NEBNext Ultra II DNA Library Prep Kit for Illumina (New England Biolabs), sequenced on the MiSeq platform (Illumina) and analyzed with GeneRead Targeted Enrichment Exon Panel Data Analysis (Qiagen) software. The mutations identified were verified by conventional Sanger sequencing.

### Quantification of mtDNA Copy Number and Analysis of Multiple Deletions

Total DNA was isolated from fibroblast pellets or muscle biopsies with the QIAamp DNA Mini kit (Qiagen). The mtDNA copy number was quantified by real-time PCR in the ABI PRISM 7500 Sequence Detection System (Applied Biosystems). mtDNA was detected using a custom-designed TaqMan probe and primers for the 12S rRNA gene (Andreu et al., 2009), and nuclear DNA using the TaqMan RNase P Control Reagent kit (ThermoFisher) in a multiplex reaction with the TaqMan Universal PCR Master Mix with UNG (Applied Biosystems).

The presence of multiple mtDNA deletions was analyzed by long-range PCR (LPCR) (Nishigaki et al., 2004).

### Western Blot

Western blot was used to analyze the protein extracts obtained from the fibroblast mitochondria-enriched fractions using the Mitochondria Isolation Kit for Cultured Cells (Abcam) following the manufacturer’s instructions. Mitochondrial pellets were resuspended in the resuspension reagent provided by the supplier, supplemented with a protease inhibitor cocktail (cOmplete EDTA-free, Roche). Samples were heat-denatured in loading buffer (60 mM Tris–HCl pH6.8, 20% glycerol, 20% SDS, 5% β- mercaptoethanol, 0.05% bromophenol blue) and separated by 12% SDS-PAGE. Proteins were transferred to Immun-Blot PVDF membranes (Bio-Rad) and detected using primary mouse monoclonal antibodies against human RNase H1 protein (ab56560, Abcam) and rabbit polyclonal antibodies against succinate dehydrogenase (SDHA; NB-22-14256, Neo Biotech).

### Recombinant Human RNase H1 Protein Production

We produced 4 different plasmids containing the following *RNASEH1* cDNA sequences: single mutant c.487T>C [p.(Tyr163His)], single mutant c.258_260del [p.(Gln86del)], double mutant c.487T>C/c.258_260del [p.(Tyr163His)/ p.(Gln86del)] and wild-type (WT). We obtained the various RNase H1 proteins as previously described (Cerritelli and Crouch, 1998). Briefly, total RNA was extracted from patient and control fibroblasts using the RNeasy Mini kit (Qiagen). The double mutant and WT *RNASEH1* cDNAs were generated using the High-capacity cDNA Reverse Transcription kit (Applied Biosystems), amplified by specific PCR using the Expand High Fidelity PCR System (Roche) with a specific pair of primers (forward 5′-ATGTTCTATGCCGTGAGGAG-3′ and reverse 5′-TCAGTCTTCCGATTGTTTAGC-3′) and cloned into the pCR 2.1 TOPO TA vector (Invitrogen). The single mutant cDNAs were obtained by site-directed mutagenesis of the WT cDNA using the Q5 Site-Directed Mutagenesis kit (New England Biolabs). Two pairs of primers were used to introduce the c.487T>C point mutation (forward 5′-AATCGGCGTTCACTGGGGGCCA-3′ and reverse 5′-CCTGCTCGCGGCCTTCTACGC-3′) and the c.258_260del deletion (forward 5′-TGGACAAGAATCGGAGGCGAAA-3′ and reverse 5′-TGATTTTCATGC CCTTCTGAAACTTCC-3′). We confirmed the absence of non-specific mutations by direct Sanger sequencing.

Each *RNASEH1* cDNA (WT, double mutant, mutant c.487T>C, and mutant c.258_260del) was subcloned into the pET-15b expression vector (Novagen) and transformed into *Escherichia coli* One Shot BL21(DE3) pLysS chemically competent cells (Invitrogen). Recombinant protein synthesis was induced in the bacterial cell culture with 1 mM IPTG, collected after cell lysis and purified using the Ni-NTA Fast Start Kit (Qiagen) based on histidine tag affinity columns.

### RNase H1 Activity Assay

The RNase H1 activity assay is based on the capability of recombinant protein to degrade RNA using a radiolabeled RNA-DNA heteroduplex as substrate (Wu et al., 2001; Alla and Nicholson, 2012). For heteroduplex generation, we labeled the 5′-end RNA oligo with ^32^P using [ϒ^-32p^]ATP. The labeling reaction was performed by incubating 200 pmol RNA oligonucleotide (5′-GAAAUACGGUCCGAAACGUUG-3′) with 3 pmol [ϒ^-32p^]ATP (3000 Ci/mmol), 290 pmol ATP, and 10 units of T4 polynucleotide kinase in 25 μl of T4 buffer (10 mM MgCl_2_, 5 mM DTT and 70 mM Tris–HCl, pH 7) for 1 h at 37°C. The ^32^P-labeled RNA oligonucleotide was then purified with Quick Spin Columns for Radiolabeled RNA Purification (Roche). For RNA/DNA heteroduplex annealing, 1 μM ^32^P-labeled RNA was incubated with 100 μM DNA oligonucleotide (5′-CAACGTTTCGGACCGTATTTC-3′), 0.5 M KCl, 250 mM HEPES, and 0.1M EDTA at 90°C for 5 min, slowly cooled-down to 37°C and then placed on ice.

The enzyme activity assay was performed by incubating 250 nM ^32^P-labeled DNA/RNA hybrid with 40 nM of recombinant RNase H1 protein and reaction buffer (0.5 M KCl, 500 nM DTT, 250 nM HEPES and 500 μM MgCl_2_) for 1 h at 30°C. The reaction was stopped by adding an equal volume of stopping solution [95% (v/v) formamide, and 20 mM EDTA]. Samples were heated at 90°C for 2 min and then resolved on 15% polyacrylamide gel with 7 M urea and TBE buffer. The gel was dried and the labeled bands were visualized by autoradiography.

### Statistics and Quantification

Data were analyzed using the GraphPad PRISM software (GraphPad software) and are presented as the mean ± SD. The protein and radiolabeled RNA bands were quantified by densitometry using ImageJ software (NIH).

## Results

### Case Report

**FIGURE 1:**
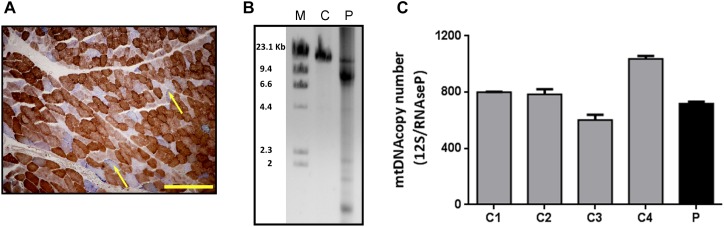
Histological and molecular findings in muscle biopsy specimens. **(A)** COX/SDH double staining of the patient’s muscle biopsy. The yellow scale bar represents 200 μm. Arrows show COX-negative fibers. **(B)** LPCR performed in DNA from the patient (P) and control (C) muscle samples. After electrophoresis, mtDNA deletions are seen as multiple bands in the agarose gel. **(C)** mtDNA copy number measured by qRT-PCR in skeletal muscle samples from patient (P) and controls (C1, C2, C3, and C4). Results are expressed as the ratio between the 12S mtDNA gene and the RNase P nuclear gene. Bars represent the mean (+SD, *n* = 3).

A 53-year-old patient was consulted for progressive palpebral ptosis without diplopia of 2 years duration, associated with a feeling of early fatigue with exercise, mild dysphagia and dysarthria. Physical examination showed moderate, fatigue-unrelated palpebral ptosis, associated with limitations in bilateral ocular movement. No significant muscle weakness was observed, except for a slight lingual weakness. The patient had slightly elevated serum creatine kinase concentration (<500 U/L). Examination of biceps muscle biopsy showed some ragged red fibers and abundant COX-negative fibers, consistent with mitochondrial myopathy (Figure 1A). No fiber size variability or central nuclei were seen. LPCR of DNA extracted from muscle biopsy revealed multiple deletions (Figure 1B) suggestive of mtDNA instability syndrome, and real-time PCR showed normal mtDNA copy number (Figure 1C).

Three years after the first visit there was little disease progression, with the exception of respiratory muscle weakness requiring non-invasive nocturnal ventilation.

### Genetic and Molecular Studies

**FIGURE 2:**
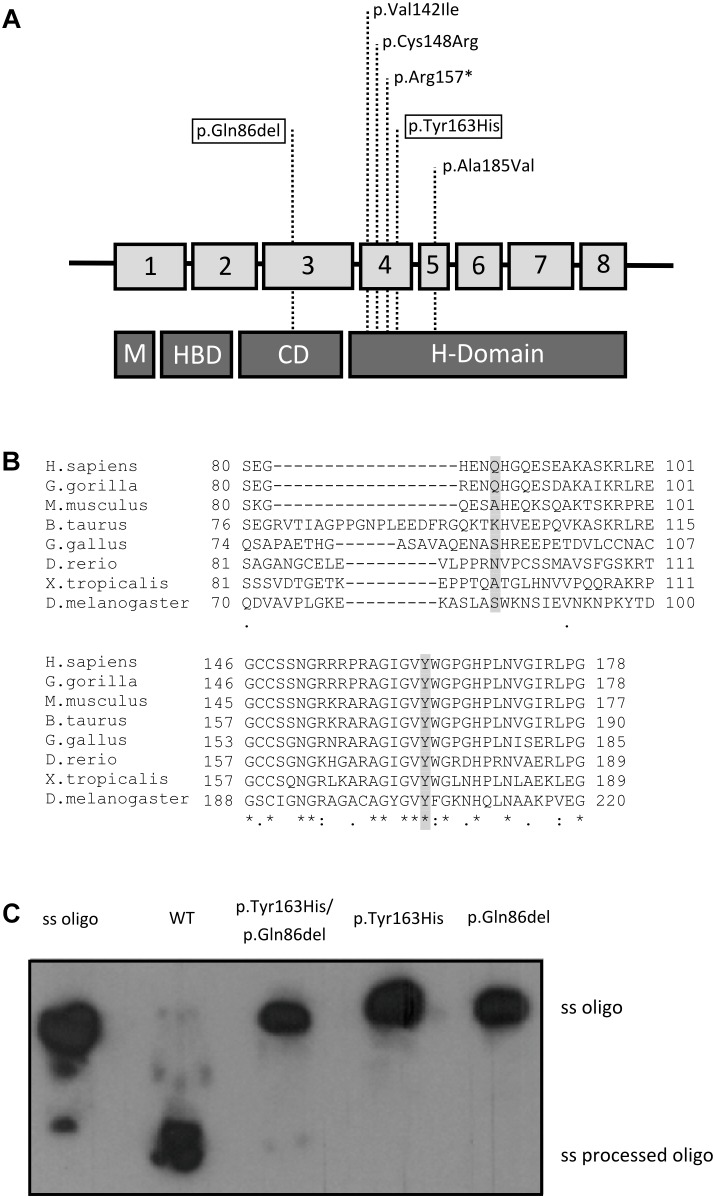
Genetic and enzymatic study. **(A)** Summary of the known *RNASEH1* mutations. Location of the mutations in *RNASEH1* and their distribution along the four different RNase H1 protein domains are indicated with dashed lines (M, mitochondrial targeting sequence; HBD, hybrid binding domain; CD, connection domain; H-Domain, catalytic domain). The two new variants are boxed within the highlighted squares. **(B)** Amino acid conservation study. CLUSTAL online software amino acid alignment of the RNase H1 regions containing the new variants. The patient’s mutated residues are shadowed and the conserved residues are indicated with an asterisk. **(C)** Enzyme activity of the wild type (WT), double mutated (p.Tyr163His/p.Gln86del), p.Tyr163His, and p.Gln86del recombinant RNase H1 proteins. The ssOligo corresponds to the uncut RNA chain of the DNA:RNA heteroduplex, whereas the ss processed Oligo is the digested RNA oligomer.

DNA sequencing from muscle biopsy specimens with the custom panel V2 (Qiagen) disclosed 2 potentially pathogenic variants in homozygosis in the *RNASEH1* gene: c.258_260del, a three-nucleotide in-frame deletion in exon 3 causing a glutamine residue deletion [p.(Gln86del)], and c.487T>C, a single nucleotide change in exon 4 causing a missense mutation [p.(Tyr163His)] (NM_002936.3, NP_002927.2) (Figure 2A,B).

First, we analyzed the population frequency of the variants, and found that both were extremely rare; c.258_260del was found in only 35 of 276096 alleles (0.013%), none in homozygosis in the gnomAD database, and the c.487T>C variant had not been previously reported in the databases consulted. To study the implication of these mutations in the patient’s phenotype, we performed an amino acid residue conservation study and *in silico* prediction of pathogenicity. The conservation studies showed that the glutamine residue at position 86 in RNase H1 protein is only conserved in primates, whereas the tyrosine residue at position 163 and the surrounding region was conserved in all species analyzed (Figure 2B). The *in silico* pathogenicity studies performed with on-line open tools classified the c.487T>C variant as “probably damaging” by Polyphen (score 1), “damaging” by SIFT (score 0) and “disease causing” by Mutation Taster. For the c.258_260del, Mutation Taster classified the variant as a polymorphism and the Alamut splicing predictor did not detect an alternative splicing, although it classified the variant as of “unknown pathogenicity.” No results were obtained with the other tools, as these predictors are unable to classify small deletions or insertions.

We measured RNase H1 enzyme activity with an *in vitro* assay using a DNA/RNA hybrid oligonucleotide as substrate and the wild-type or mutated recombinant proteins containing each single mutation or a combination of both. Only the wild-type recombinant protein displayed RNase H1 activity. No residual activity was detected for any mutated recombinant protein (Figure 2C).

### Characterization of the Mutation in Skin Fibroblasts

**FIGURE 3:**
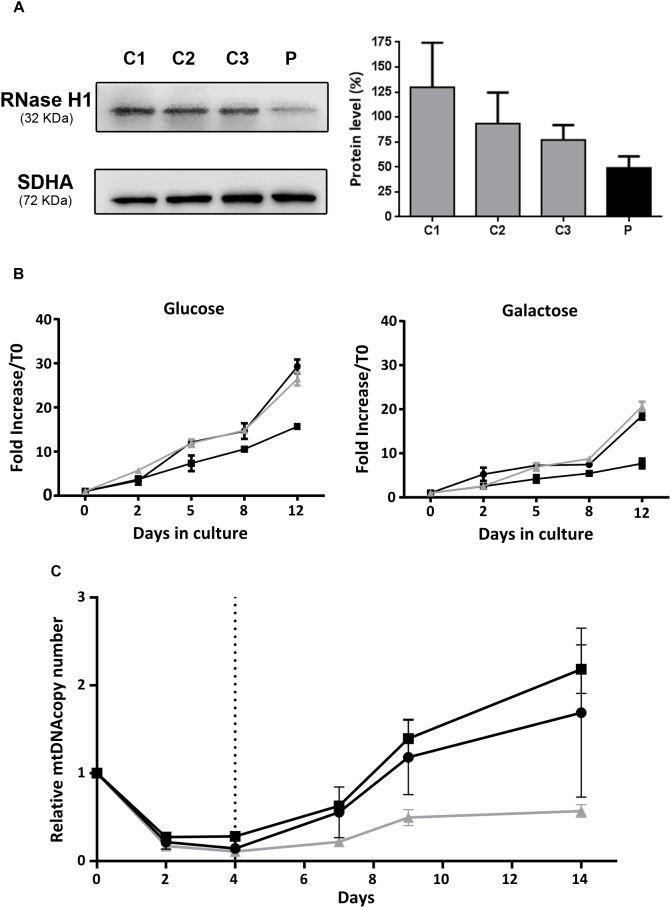
Functional studies in patient fibroblasts. **(A)** Western blot analysis of RNase H1 in mitochondria-enriched protein extracts from patient (P) and control (C1, C2, and C3) fibroblasts. The bar chart represents relative quantification of RNase H1 protein by densitometry. Results are expressed as the percentage of the mean (+SD, *n* = 3) RNase H1 protein density value obtained with control fibroblasts corrected with the SDHA protein load control. **(B)** Cell growth curves of fibroblasts incubated at 37°C in culture media supplemented with glucose (4.5 g/L) or galactose (1 g/L) during 12 days. The black lines correspond to control fibroblasts and the gray line to patient fibroblasts. Results are expressed as the mean fold increase with respect to the total cell count at time 0 (±SD, *n* = 3). **(C)** Recovery from mtDNA depletion in fibroblasts. Control (black lines) and patient (gray line) fibroblasts were incubated at 37°C in complete culture media supplemented with EtBr (15 ng/mL) during 4 days. EtBr was removed from culture media (dashed vertical line) and cells were maintained in culture during 10 additional days. DNA was extracted on days 0, 2, 4, 7, 9, and 14, and mtDNA copy number was quantified by qRT-PCR as the ratio between the 12S and RNase P gene copy numbers. Results are expressed as the mean mtDNA copy number referred to day 0, for each cell line (±SD, *n* = 2).

RNase H1 steady state levels were measured in skin fibroblast mitochondrial protein extracts from the patient and a controls. Western blot analysis showed a marked RNase H1 reduction in the patient’s sample: residual protein level was only 49% of the mean levels in control fibroblasts (Figure 3A).

We compared the capacity for growth of patient fibroblasts and two control cell lines. Cells were cultured in medium with glucose for 12 days with regular medium changes, and cell counts were performed at several time points. No differences in growth were observed between the patient and control cell lines (Figure 3B). In order to force cells to preferentially use oxidative phosphorylation for ATP production, we grew cells in culture medium supplemented with galactose instead of glucose, and observed an expected growth rate reduction in all cell lines, with no differences between control and patient cells (Figure 3B).

Mitochondrial DNA content and integrity was analyzed in patient fibroblasts, but neither deletions nor mtDNA depletion were detected. As the fibroblasts did not show a spontaneous mtDNA phenotype, we induced mtDNA depletion in samples from the patient and two controls. To this end, we treated fibroblasts with EtBr and then assessed their ability to recover normal mtDNA copy number. mtDNA copy number had decreased at 2 and 4 days after EtBr treatment in all fibroblast lines (Figure 3C). At 5 days after EtBr removal, control fibroblasts had recovered the initial mtDNA copy number, which continued to increase up to the end of the experiment (10 days), whereas patient fibroblasts were unable to recuperate, showing mtDNA copy number at only 60% (Figure 3C).

## Discussion

Mitochondrial depletion and deletion syndromes are a group of mitochondrial disorders caused by mutations in nuclear genes associated with mtDNA instability. These conditions have a wide variety of clinical presentations ranging from a spectrum of severe early onset neurologic or other organ-specific syndromes to adult-onset milder encephalomyopathies characterized by PEO (Viscomi and Zeviani, 2017). One possible genetic cause of the milder clinical form is the presence of mutations in *RNASEH1*, an uncommon occurrence with only 14 patients reported to date, all of them showing PEO as a clinical trait (Reyes et al., 2015; Bugiardini et al., 2017). Here, we report the case of a patient with PEO and additional clinical features commonly reported in other patients with *RNASEH1* mutations, such as dysphagia, dysarthria, respiratory impairment, and slight lingual muscle weakness (Bugiardini et al., 2017). Histological and molecular studies in skeletal muscle found ragged red fibers, COX-defective fibers, and multiple mtDNA deletions, which are always present in patients with *RNASEH1* mutations. Although the clinical features of our patient matched many of those reported in others with *RNASEH1* mutations, the low prevalence of the disease and the large number of genes associated with these symptoms hampered the diagnosis.

Our patient showed two mutations in *RNASEH1*, detected by high-throughput sequencing using a custom panel of genes involved in mtDNA replication and maintenance. High-throughput sequencing techniques have improved the diagnosis of mitochondrial diseases. In fact, all reported *RNASEH1* mutations have been detected using target or whole-exome sequencing (Reyes et al., 2015; Bugiardini et al., 2017). The panel used in this study provided a fast and cost-effective way to establish the genetic diagnosis in a patient with a specific MDDS. However, the number of genes known to be involved in these phenotypes is growing, and next-generation sequencing techniques, such as whole exome sequencing, are sometimes needed to reach a definite genetic diagnosis.

Maturation of mtDNA during replication involves RNase H1 and at least two additional proteins encoded by the *MGME1* and *DNA2* genes, which are also implicated in MDDSs with PEO (Kornblum et al., 2013; Ronchi et al., 2013; Viscomi and Zeviani, 2017). RNase H1 protein is essential for terminating replication by removing the replication primer, and defects in its function cause deceleration and stalling of mtDNA replication (Reyes et al., 2015). Here, we investigated the molecular effects of *RNASEH1* mutations on the patient’s fibroblasts and found a marked decrease in RNase H1 protein in mitochondria, indicating that one or both variants affected protein stability. However, we did not observe multiple deletions or mtDNA depletion, which likely explains the normal growing rate seen in the OXPHOS-obligatory culture conditions. This limitation of the model, commonly observed in cultured fibroblasts from other MDDSs, is usually bypassed by assessing the ability to restore normal mtDNA copy number following induced mtDNA depletion (Stewart et al., 2011; Kornblum et al., 2013; Dalla Rosa et al., 2016). In our case, fibroblast mtDNA recovery was clearly reduced, thus demonstrating that the pathogenesis of the disease was related to an mtDNA replication defect, in agreement with other cases involving *RNASEH1* mutations (Holmes et al., 2015; Reyes et al., 2015).

The *RNASEH1* mutations found were homozygous and neither had been previously described as disease-related. Genetic study of the mutations in the patient’s parents was not possible, but we know that they were consanguineous, which reduces the possibility of apparent homozygosity due to compound heterozygosity of the two mutations with an overlapping long-range gene deletion (Landsverk et al., 2012; Martins Rda et al., 2014; Shibata et al., 2015). We also knew that neither of the patient’s parents were affected, which concurs with the previously reported autosomal recessive inheritance of the disease (Reyes et al., 2015; Bugiardini et al., 2017).

*In vitro* study of recombinant protein function carrying the two mutations demonstrated a complete loss of activity, which points to them as the cause of the disease. Our functional studies also showed that each mutation separately resulted in complete loss of RNase H1 activity, suggesting that either mutation alone can cause the disease. These results were partially consistent with our conservation study and our *in silico* prediction of pathogenesis, since all results indicated that the missense mutation located in the catalytic domain was pathogenic. However, pathogenicity prediction of the deletion located in the connection domain did not concur with the results obtained from the functional study. One potential explanation is that the deletion was predicted to be a polymorphism or of unknown pathogenicity because it was found in the population in heterozygosis at a frequency of 0.01% (gnomAD database). Moreover, only two of the four predictors used were suitable for assessing small deletions and their predictions are based, among other factors, on the mutation frequency in population databases, evolutionary conservation, splice-site changes, and loss of protein features (Schwarz et al., 2014), thus highlighting the importance of using various pathogenic predictors and functional validation to study new variants. Finally, both variants can be classified as “likely pathogenic” following the American College of Medical Genetics and Genomics (ACMG) guidelines for the interpretation of sequence variants because c.478T>C meets the pathogenicity criteria PS3, PM1, PM2, and PP3 and c.258_260del meets the pathogenicity criteria PS3 and PM4 (Richards et al., 2015).

## Conclusion

In conclusion, our data support the pathogenicity of the two *RNASEH1* variants found in a patient with PEO syndrome, multiple deletions, and mild mitochondrial myopathy, phenotypic traits that are characteristic of other patients with mutations in the same gene.

## Data Availability

The raw data supporting the conclusions of this manuscript will be made available by the authors, without undue reservation, to any qualified researcher.

## Ethics Statement

All the experimental protocols were performed with appropriate informed consent and approval of the Clinical Research Ethics Committee of the Hospital Universitari Vall d’Hebron (PR(IR)66/2016).

## Author Contributions

LC-G, CB-B, JD-M, EG, and JT-T acquired the data. LC-G, CB-B, JD-M, EG, YC, RM, JT-T, and EG-A analyzed and interpreted the data. LC-G, JD-M, EG, JT-T, and EG-A drafted the manuscript. LC-G, YC, RM, JT-T, and EG-A critically revised the manuscript for important intellectual content. LC-G, JT-T, and EG-A approved the final version of the manuscript to be published.

## Conflict of Interest Statement

The authors declare that the research was conducted in the absence of any commercial or financial relationships that could be construed as a potential conflict of interest.
